# Metabolomics Strategy Using High Resolution Mass Spectrometry Reveals Novel Biomarkers and Pain-Relief Effect of Traditional Chinese Medicine Prescription Wu-Zhu-Yu Decoction Acting on Headache Modelling Rats

**DOI:** 10.3390/molecules22122110

**Published:** 2017-12-18

**Authors:** Ran Liu, Huarong Xu, Xiaowen Zhang, Xiaotong Wang, Ziyue Yuan, Zhenyu Sui, Dong Wang, Kaishun Bi, Qing Li

**Affiliations:** 1 National and Local Joint Engineering Laboratory for Key Technology of Chinese Material Medica Quality Control, School of Pharmacy, Shenyang Pharmaceutical University, 103 Wenhua Rd., Shenyang 110016, China; liuran8515@hotmail.com (R.L.); huarongxu@outlook.com (H.X.); wangxt61@163.com (X.W.); yzy_127@sina.com (Z.Y.); kaishunbi.syphu@gmail.com (K.B.); 2 Guangzhou Bristol Drug Delivery Co., Ltd., 11 Kaiyuan Ave, Guangzhou 510320, China; xiaowen.zhang@bristoldrug.com; 3 China Food and Drug Administration Institute of Executive Development, 16 Xi Zhan Nan Rd., Beijing 100073, China; iamdanielsui@126.com; 4 Dalian Institute for Drug Control, 888a Huanghe Rd., Dalian 116000, China; wd13591146956@126.com

**Keywords:** headache, high resolution mass spectrometry, metabolomics, traditional Chinese medicine, Wu-Zhu-Yu decoction

## Abstract

Headache is a common episodic or chronic neurologic disorder. Treatment options and diagnosis are restricted by an incomplete understanding of disease pathology and the lack of diagnostic markers. Wu-Zhu-Yu decoction (WZYD), a traditional Chinese medicine (TCM) formula containing four TCM herbs, is commonly used in the treatment of headache in China. To deeply understand more about headache and investigate the pain-relief mechanism of WZYD, a comprehensive metabolomics study combined with multivariate data processing strategy was carried out. An LC-high resolution mass spectrometry-based metabolomics approach was applied to characterize metabolic biomarker candidates. Multiple pattern recognition including principal component analysis-discriminant analysis, partial least squares-discriminant analysis and hierarchical cluster analysis were used to determine groups and confirm important variables. A total of 17 potential biomarkers were characterized and related metabolic pathways were identified. The study demonstrated that the established metabolomics strategy is a powerful approach for investigating the mechanism of headache attack and WZYD. In addition, the approach may highlight biomarkers and metabolic pathways and can capture subtle metabolite changes from headache, which may lead to an improved mechanism understanding of central nervous system diseases and TCM treatment.

## 1. Introduction

Wu-Zhu-Yu decoction (WZYD) is a traditional Chinese medicine (TCM) formula for the treatment of headache and gastrointestinal disorders. The formula was originally recorded in Shang Han Lun, which is a classic TCM book written in the Eastern Han Dynasty of ancient China. The formula contains four TCM herbs: Euodiae Fructus (dried and nearly ripe fruit of *Euodia rutaecarpa* (Juss.) Benth.), *Zingiberis Rhizoma Recens* (dried rhizome of *Zingiber officinale* (Willd.) Rosc.), *Ginseng Radix et Rhizoma* (dried root or rhizome of *Panax ginseng C. A. Mey*.) and Jujubae Fructus (the dried ripe fruit of *Ziziphus jujube Mill*.). WZYD has currently been used for the treatment of headache, nausea, gastrointestinal disorders, dysentery and postpartum hemorrhage, etc. with high efficiency and low toxicity in clinical application in China [1].

Headache is a disabling neurologic condition that affects a considerable amount of the general population and is accompanied with phonophobia, nausea and vomiting [2,3]. Headache occurs in migraines, tension-type headaches, and cluster headaches [4]. Frequent headaches can affect relationships and employment, and there is also an increased risk of depression in those with severe headaches [4]. The specific pathogenesis and mechanism of headache is not exact, and there are also some new hypothesis and studies being put forward currently. Some researchers believe neuronal mechanisms play a greater role [5], while others believe blood vessels play the key role [6]. We do not completely understand the pathogenesis of pain in headache, but three key factors merit consideration: the cranial blood vessels, the trigeminal innervation of the vessels, and the reflex connections of the trigeminal system with the cranial parasympathetic outflow [7]. Abnormal levels of small molecule metabolites—for instance, neurotransmitters like serotonin—are believed to be involved [8].

Study on the biochemistry of headache began as early as the 1960s and was primarily directed at serotonin metabolism after an increase of 5-hydroxyindoleacetic acid (5-HIAA), the main metabolite of serotonin, which was observed in the urine of migraine sufferers [9]. Genetic and biochemical studies have primarily focused on the neurotransmitter serotonin, considering receptor binding, transport and synthesis of serotonin and have investigated serotonergic mediators including enzymes, receptors as well as intermediary metabolites. These studies have been mainly assayed in blood, cerebral spinal fluid and urine as the most accessible fluids [9].

Metabolomics is an “-omics” science in systems biology, providing a powerful analytical strategy in biomarker discovery and TCM study, demonstrating significant potential in many fields such as disease diagnosis, natural product discovery, and toxicology, etc. [10,11]. While genomics and proteomics suggest a possible mode of operation of the system, metabolomics, which focuses on small molecule metabolites, gives the actual representation of the system [12]. In agreement with the multifactorial nature of the disease, the studies conducted to date relate to different classes of analytes. Liquid chromatography-high resolution mass spectrometry (LC-HRMS) has become a preferred strategy in TCM component and metabolomics study on account of its rapid, high separation efficiency, high accuracy, high resolution, sensitive, large-scale dynamic range and excellent generality [13,14]. Common mass analyzers of high resolution mass spectrometer include time-of-flight (TOF), orbitrap and FT-ICR. However, despite the fact that FT-ICR and orbitrap have higher resolution and mass accuracy, the TOF analyzer possesses a much higher scanning rate, which is more suitable in the complex TCM system and biofluids.

Therefore, in this study, an LC-HRMS-based metabolomics approach was developed to characterize the metabolic biomarkers on headache, and to discover potential targets of WZYD on headache treatment. Sensitive and specific potential biomarkers were eventually characterized, while a pattern recognition approach was carried out to estimate the changes of metabolite levels in brain and plasma and to identify headache biomarker candidates. In addition, the study described the discovery and development of WZYD on headache, and then provided a scientific example to highlight the power of the LC-HRMS system for sophisticated biological data sets.

## 2. Results

### 2.1. Behavior Study Results

Classic animal headache modelling induced by nitric oxide (NO)-donor nitroglycerin (NTG) was adopted and established in the study. The pharmacological behavior of the animals demonstrated the succeed of headache. Three minutes after the injection of NTG, the rats in the model group, WZYD group and flunarizine group showed the phenomena of head-twitch, ear rosacea and forelimb scratching head frequently, which lasted throughout the 2 h observation. In contrast, only 0–10 times head-scratching showed up in the rats of the control group occasionally.

The results of statistical analysis between each group using the independent samples *t*-test are listed in Table S1. The success of modeling was obviously demonstrated by the statistical results through the significant differences between the control and model group (*p* < 0.05), which demonstrated a significant increase of the times of head-scratching. Comparing to the model group, the times of head-scratching of the rats in WZYD group and flunarizine group decreased obviously, which indicated the pharmaceutical effect of WZY decoction and flunarizine on headache through the statistically significant differences between the two groups (*p* < 0.05).

### 2.2. Multivariate Statistical Analysis of Metabolite Profiling

**Figure 1:**
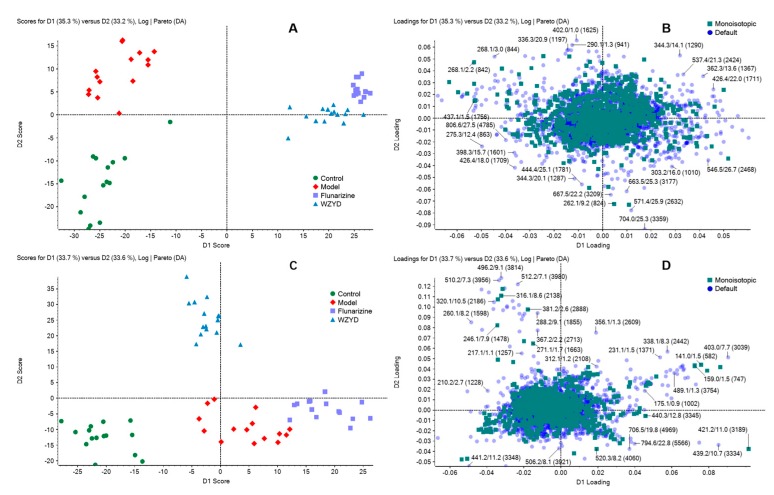
The principal component analysis-discriminant analysis (PCA-DA) recognition based on the brain homogenates and plasma metabolomic profiling. (**A**) Brain metabolomics score plot of control group (green), model group (red), Wu-Zhu-Yu decoction (WZYD) group (blue) and flunarizine group (purple); (**B**) Loading plot, each dot represents a compound which contributed in group separation; (**C**) Plasma metabolomics score plot; (**D**) Plasma metabolomics loading plot.

Fast scan information dependent acquisition (IDA) was applied in the LC-HRMS detection. Typical chromatograms of brain tissue and plasma are shown in Figure S1. Chromatographic peaks were then carried out for alignment and normalization, followed by multivariate statistical analysis. Principal component analysis-discriminant analysis (PCA-DA), a supervised multivariate analysis comparing with PCA, was employed for metabolomics study to differentiate among the groups. As shown in the PCA-DA score plots, both the brain tissue and plasma samples from the control, model, WZYD and flunarizine group were well separated into four categories (Figure 1), suggesting that metabolic perturbation significantly occurred after modelling as well as the WZYD and flunarizine treatment.

### 2.3. Metabolite Identification

High resolution QTOF (quadrupole time-of-flight) MS information was acquired through the LC-HRMS, and all significantly differentiated metabolites satisfying corrected *p*-value cut-off 0.05 in one-way ANOVA were listed. A series of matched results were then obtained after online database searching including mzCloud and METLIN with mass error threshold set as 5 ppm. After the LC separation and HRMS detection, 4484 and 4842 chromatographic peaks were detected in brain and plasma metabolomics study, and 1181 and 1656 metabolites are with statistically significance between the control and model group, respectively.

**Figure 2:**
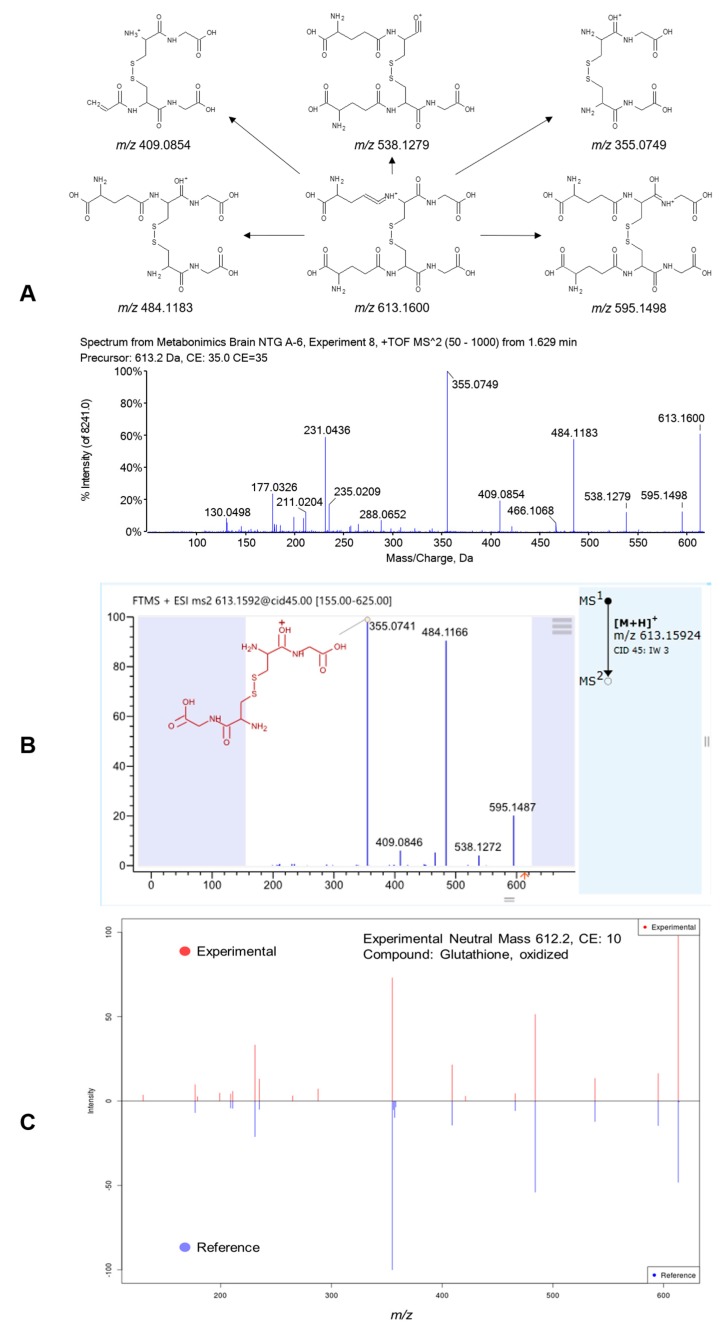
(**A**) Fragmentation pathway and product ion spectrum of oxidized glutathione; (**B**) MS/MS spectrum of oxidized glutathione by mzCloud and (**C**) the comparison spectrum between the experimental and reference MS/MS spectrum by METLIN.

According to the accurate *m*/*z*, fragmentation pathway, chromatographic retention behavior, and comparison with database and reference standards, 12 potential metabolic biomarkers were identified after brain tissue metabolomics screening, including glycerophosphocholine (GPC), arginine, adenosine monophosphate (AMP), nicotinamide adenine dinucleotide (NAD), glutathione (GSH), oxidized glutathione (GSSG), adenine, adenosine, valerolactam, butyrylcarnitine, caprolactam and cholecalciferol (also known as vitamin D3). The information of the potential biomarkers in HMDB, MTLIN and KEGG and their trends in headache rat brain tissue are listed in Table S2. Retention time, *m*/*z* and metabolite intensities of the potential brain homogenates biomarkers in control, model, WZYD and flunarizine groups analyzed by one-way ANOVA are listed in Table 1. Fragmentation pathway, product ion HRMS spectrum, standard spectrum in mzCloud and the comparison spectrum between the experimental and reference MS/MS spectrum by METLIN of a representative metabolite, oxidized glutathione, are shown in Figure 2. Results indicated that the level of arginine and cholecalciferol in brain tissue down-regulated, and inversely, the level of other 10 potential markers up-regulated. After the treatment of WZYD, the level of all 12 biomarkers in rat brain significantly back-regulated comparing with the model group.

Using the same screening pattern as the brain tissue, a total of 6 potential metabolic biomarker were identified after plasma metabolomics screening, including arginine, carnitine, 5-hydroxyindoleacetic acid (5-HIAA), 3-methylindole, hydroxyindole and indoleacetic acid (IAA). Relative information of the 6 metabolites are listed in Table 2 and Table S3. Extracted ion chromatograms of the potential metabolite biomarkers found in both brain and plasma metabolomics are shown in Figure S2. Results indicated that the level of all 6 metabolites in rat plasma down-regulated. After the treatment of WZYD, the level of arginine, carnitine and 3-methylindole in rat plasma significantly back-regulated comparing with the model group.

### 2.4. Differential Metabolites Related to Headache

**Figure 3:**
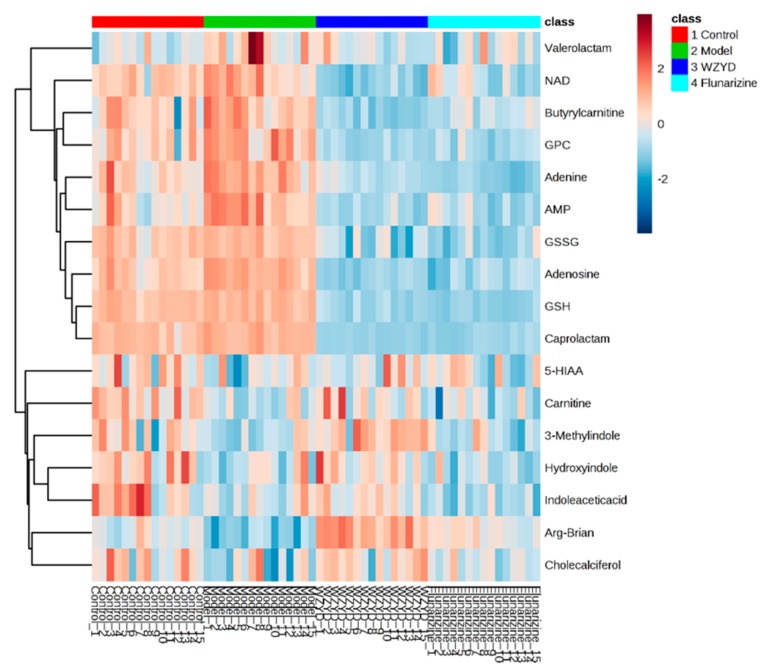
The hierarchical clustering heatmap of the potential biomarkers.

The pattern recognition analysis of partial least-squares-discriminant analysis (PLS-DA) on the biomarker candidates was employed after metabolites characterization, and the contribution of each potential biomarkers to the discrimination among the groups were ranked as VIP scores shown in Figure S3. The heatmap (Figure 3) using MetaboAnalyst 3.0 [15,16] demonstrated different distribution patterns of totally 17 potential biomarkers among the four groups, and moreover, the results of hierarchical cluster analysis (HCA) provided a distinct visualization of the groups.

### 2.5. Pathway Analysis and Metabolic Network

**Figure 4:**
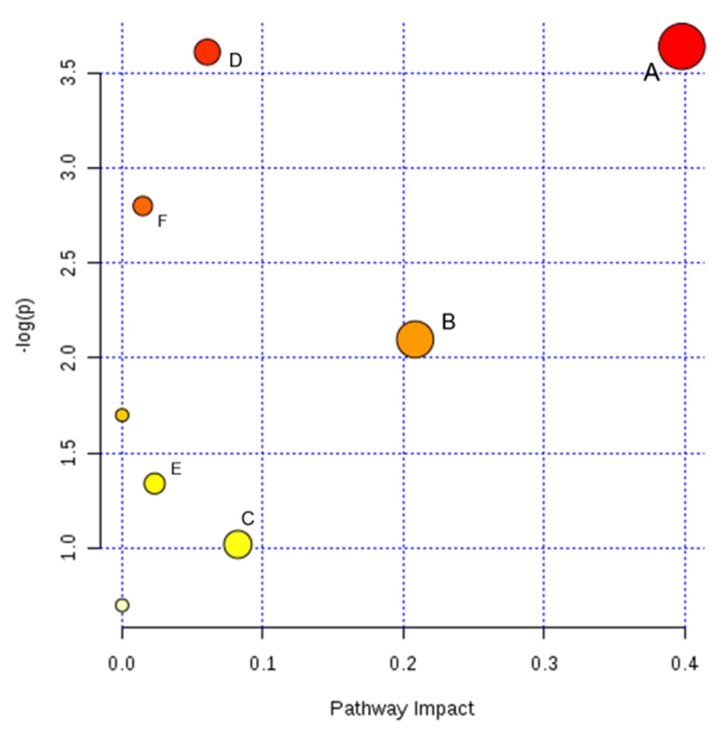
The metabolic pathways related to headache, as analyzed by MetaboAnalyst. The map was generated using the 379 reference map by KEGG. (A) Glutathione metabolism; (B) Nicotinate and nicotinamide metabolism; (C) Arginine and proline metabolism; (D) Purine metabolism; (E) Glycerophospholipid metabolism; (F) Tryptophan metabolism.

The metabolic pathways related to the identified potential biomarkers were estimated by pathway topology analysis using MetaboAnalyst based on the KEGG and SMPD reference pathways, in order to find possible metabolic pathways that have great impact on headache. The searching results of the pathways correlative with the 17 candidate markers are listed in Table S4, while Table 3 shows the results of pathway topology analysis on the potential markers. A figure of metabolic pathway impact which demonstrated the pathway impact values is shown in Figure 4. Results indicated that glutathione metabolism, nicotinate and nicotinamide metabolism, arginine and proline metabolism, purine metabolism, glycerophospholipid metabolism and tryptophan metabolism would be considered closely related to the attack of headache (impact > 0.01). Consequently, based on the level of the back-regulation on biomarkers after the WZYD treatment, glutathione metabolism, arginine and proline metabolism and purine metabolism were presumed as the pathways related to the headache-relief effect of WZYD.

Total is the total number of compounds in the pathway; the Hits is the actually matched number from the user uploaded data; the Raw *p* is the original *p* value calculated from the enrichment analysis; the Impact is the pathway impact value calculated from pathway topology analysis.

The identified potential metabolic biomarkers and their corresponding pathways were imported into the Cytoscape software for visualization of the metabolites interaction network, which is shown in Figure S4. Altered metabolites were mapped to KEGG and SMPD reference pathways and interaction networks were generated. The network nodes (blue and pink) represent pathways and related metabolites detected, respectively. The networks would help to understand in-depth the pathobiological mechanism of headache attack.

## 3. Discussion

### 3.1. Characterized Potential Biomarkers

**Figure 5:**
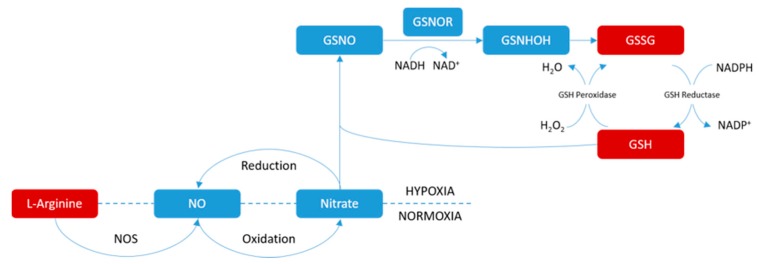
Part of the nitric oxide signaling pathway.

Metabolic changes are relevant to many diseases. A group of biomarkers used to characterize disease could be helpful to understand the key features of headache, and be useful for the prevention, diagnosis, and treatment of headache. Arginine is a basic amino acid, and l-arginine is the form with bioactivity. It is not only a conditional essential (also known as semi-essential) amino acid which plays an important role in the biosynthesis of proteins, but the precursor of a number of bioactive compounds, such as polyamines and NO. Arginine is the only biosynthetic substrate of NO in vivo, while the nitric oxide signaling pathway plays an essential role in the central nervous system (CNS) (Figure 5). Our study listed arginine as a biomarker candidate in both brain and plasma metabolomics screening, and both showed down-regulation. A possible explanation is the headache animal model adopted in the study, which was the NO-donor model, while the nitroglycerin could release a considerable quantity of NO, which leads to the vessel’s over expansion, and eventually the experimental headache attack was triggered. Large amounts of exogenous NO might inhibit the synthesis of endogenous NO and lead to a down-regulation of its precursor, arginine. In the meantime, the increased level of arginine might lead to an up-regulation of its downstream metabolites, GSH and GSSG. Gökçe et al. compared the oxidative and anti-oxidative level between the serum samples of headache patients and healthy volunteers, and the study demonstrated the activity of GSH peroxidase in headache patients were significantly stronger than that in normal people, which also proved that GSH and GSSG in headache patients became more active [17].

**Figure 6:**
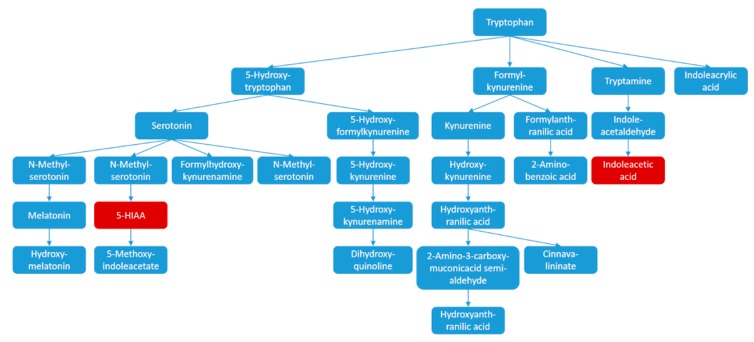
Tryptophan metabolism pathway.

Tryptophan metabolism pathway (Figure 6) includes the metabolism of a series of monoamine neurotransmitters, such as tryptophan and its downstream metabolites 5-hydroxytryptamine (5-HT, serotonin), 5-hydroxy indole acetic acid (5-HIAA), melatonin, kynurenic acid and quinolinic acid, etc. Tryptophan metabolism pathway has a prominent position in the pathologic process of neurodegenerative diseases, and acts through various regulations. A good example is the serotonin level in headache patients, which was typically abnormal [18,19]. Kynurenic acid has the effect of protection on CNS, while kynurenine and quinolinic acid could be neurotoxic [20,21]. Among the neurotransmitters in tryptophan metabolism pathway, 5-HT was the most studied and reported on headache. However, the types of primary headache were diversified, while the pathogenesis and why the attack happens still remains unclear; therefore, the level of 5-HT cannot represent all the headaches. Results indicated that there was no significant difference of the 5-HT level between the control and model group; however, the level of its metabolite, 5-HIAA significantly down-regulated in plasma. Consequently, there was no direct connection between NO-donor headache model and the 5-HT level, but the metabolic disorder might lead to abnormalities in the tryptophan metabolic pathway. The down-regulation of the downstream metabolite, IAA, provided evidence for the speculation as well. Among all detected potential biomarkers, the level of 3-methyl-indole and hydroxy-indole were also down-regulated. Despite the fact that the two metabolites are not on the tryptophan pathway, according to their chemical structures, they may probably be attributed to the product compounds of the tryptophan pathway.

Adenine and adenosine, along with AMP, which participates in many bioactivities including the synthesis of DNA and RNA, are all significant parts of the purine metabolism. Adenosine plays a significant role in a number of CNS diseases [22], but no headache-related study had been reported yet. Nicotinamide adenine dinucleotide (NAD^+^, Co-enzyme I) is synthesized by adenosine and nicotinic acid. It is involved in many cellular processes, including cell signaling, DNA repair, gene regulation and apoptosis as the essential co-enzyme in oxidation-reduction. It is also the only material that can be used for NAD^+^-dependent ADP-ribosyltransferase, poly (ADP-ribose) polymerase, cyclic (ADP-ribose) polymerase and histone deacetylases 3 (HDAC3) [23,24]. In the metabolic network we built in the study, NAD^+^ and NADH were involved in many reactions, which might lead to the increase of its activity. Therefore, it was presumed that NAD^+^ and its synthetic materials would up-regulate, which is in accordance with the study result.

For the other identified potential markers, a previous study showed that low serum carnitine level might be one of the trigger of headache attack, which coincided with our study [25]. Cholecalciferol was demonstrated as down-regulated in the study, and it had been listed as a biomarker of headache in many reported studies on headache patients [26,27,28]. Clinical research indicated that 26% of the headache patients exhibited vitamin D deficiency, and the research also demonstrated that lack of cholecalciferol might signify the damage of neuromuscular functions in chronic pain patients [28].

### 3.2. Possible Mechanism of Pain-Relif Action by WZYD

The integrated metabolomics study demonstrated that WZYD acted through different ways with the positive control chemical drug flunarizine, meaning that WZYD might regulate differently for different potential biomarkers. The arginine level in both brain tissue and plasma were all significantly back-regulated after the administration of WZYD, while flunarizine exhibited the same effect only in brain homogenates. A possible explanation is that flunarizine, a selective calcium channel antagonist, exerts its therapeutic effects by inhibiting the transmembrane flux of calcium ions into cells. It has a higher selectivity on brain vessels, and lower selectivity on myocardial vessels. In brain tissue metabolomics, WZYD significantly showed back-regulation on cholecalciferol, on the contrary, flunarizine gave us an opposite result. Meanwhile, in plasma metabolomics, WZYD and flunarizine also went different ways on the tryptophan pathway. The result indicated that WZYD may possess the regulation of metabolic disorders of headache with which flunarizine works, and furthermore, WXYD may act more on arginine, cholecalciferol and the tryptophan pathway. Consequently, the result revealed that WZYD may have more effective targets, with more comprehensive actions on the relief of headache.

## 4. Materials and Methods

### 4.1. Materials and Preparation of Wu-Zhu-Yu Decoction

HPLC-grade acetonitrile, methanol, water and LC-MS-grade formic acid were all supplied by Fisher Scientific (Fair Lawn, NJ, USA). The reference standards of GSH and 5-HIAA were from Sigma-Aldridge (St. Louis, MO, USA). Nitroglycerin injection was from Beijing Yimin Pharmaceutical Co., Ltd. (Beijing, China) and flunarizine capsules were from Xian Janssen Pharmaceutical Ltd. (Xi’an, China). The four herbs in the formula, Euodiae Fructus, Zingiberis Rhizoma Recens, Ginseng Radix et Rhizoma and Jujubae Fructus were obtained from Tong-Ren-Tang TCM store (Shenyang, China) and authenticated by Dr. Ying Jia (Shenyang Pharmaceutical University, Shenyang, China). WZYD was prepared with the procedure based on the original composition and preparation method as previously described [29]. Briefly, the four herbs were boiled together in water for 2 h twice and then filtered through filter paper. The filtrate was then combined and concentrated under reduced pressure to 0.8 g crude drug per mL.

### 4.2. Animal Modelling and Treatments

Sixty male pathogen-free Sprague-Dawley rats of 6–8 weeks of age (weighing 220 ± 20 g) provided by the Experimental Animal Center of Shenyang Pharmaceutical University were raised in an SPF-level environment kept at a temperature of 22 ± 2 °C and a relative humidity of 50 ± 10%, with a natural light-dark cycle. All animals were allowed to acclimatize in cages for a week prior to treatment. After acclimatization, all the rats were randomly divided into four groups of fifteen rats each as follows: control group, model group, WZYD group and flunarizine group.

NTG of NO-donor animal headache model was adopted and established. The four groups were treated as follows: the model group, WZYD group and flunarizine group were all injected with nitroglycerin subcutaneously at a dose of 10 mg kg^−1^ to establish the headache model induced by NO donor, while the control group was injected with physiological saline subcutaneously at the same volume. Following the injection, all the rats of the four groups were placed, one at a time, in an observation cage. The behaviors and symptoms of the four groups of rats were observed and recorded continuously for 2 h. Times of head scratching was the main ethology index which was used to demonstrate the success of the modeling in this study. Statistical analysis between each two of the four groups was performed by SPSS 19.0 software (IBM, Armonk, NY, USA) using independent samples *t*-tests. A *p* value less than 0.05 was considered statistically significant for all the tests. All data were presented as means ± SD.

The WZYD group was intragastrically administrated with WZYD at a dose of 6.67 g kg^−1^ (crude drug/body weight) 20 min after the injection of nitroglycerin. The dosage for oral administration of WZYD was according to the original dose described in *Shang Han Lun*. The flunarizine group was intragastric administrated with flunarizine suspension made by the flunarizine capsules at a dose of 1 mg kg^−1^. Animal studies were carried out in accordance with the Guidelines for Animal Experimentation of Shenyang Pharmaceutical University, and the protocol was approved by the Animal Ethics Committee of the Institution (Ethic approval document No. SYPU-IACUC-C2017-1-31-203). The experimental methods were conducted according to the principles expressed in the Declaration of Helsinki.

### 4.3. Collection and Preparation of Biosamples

Blood samples (approximately 5.0 mL) were collected from the fosse orbital vein using heparinized sterile microtubes at 2 h after headache modelling. All the blood samples were centrifuged immediately at 1500× *g* for 10 min, then transferred and stored at −80 °C until analysis. Plasma samples were thawed at 4 °C before analysis. 400 μL cold acetonitrile was added into 200 μL aliquot of plasma and vortexed about 3 min for protein precipitation. The mixture was centrifuged at 13,000× *g*, 4 °C for 10 min. The supernatant was separated and dried by vacuum centrifugation. The dried residue was reconstituted with 200 μL of acetonitrile and water (2:98, *v*/*v*) followed by vortexing for 3 min, sonicating for 3 min and centrifuging at 13,000× *g* for 5 min.

After the plasma samples collection, the whole brain samples were collected immediately and rapidly. The brain samples were temporarily kept in a dry ice box and then stored at −80 °C until analysis. Brain tissue samples were thawed at 4 °C before analysis. Ten times volume of cold acetonitrile was added into the brain tissue and homogenized under ice water bath. The mixture was centrifuged at 13,000× *g*, 4 °C for 10 min. 1 mL of supernatant was dried by vacuum centrifugation. The dried residue was reconstituted with 100 μL of acetonitrile and water (2:98, *v*/*v*) followed by vortexing for 3 min, sonicating for 3 min and centrifuging at 13,000× *g* for 5 min.

The preparation and analysis of the quality control samples was shown in Supplementary Materials.

### 4.4. Liquid Chromatography-High Resolution Mass Mpectrometry Analysis

The LC separation was performed using a 1260 Infinity LC system (Agilent, Santa Clara, CA, USA), while the chromatographic separation was performed on an Agilent RRHD Zorbax Eclipse Plus C18 (100 mm × 2.1 mm, 1.8 µm, Agilent, Santa Clara, CA, USA) column at 30 °C. Analysis was completed with a gradient elution of 0.1% formic acid in water (A) −0.1% formic acid in acetonitrile (B) within 30.0 min. The gradient program was 98% A at 0.0–1.0 min; 98% A→43% A at 1.0–7.5 min; 43% A→5% A at 7.5–20.0 min; 5% A at 20.0–24.0 min; 5% A→98% A at 24.0–24.1 min; 98% A at 24.1–30.0 min at a flow rate of 0.3 mL min^−1^ with a sample injection volume of 4.0 μL. All the samples were kept at 4 °C during the analysis.

The high resolution MS system was performed using a Sciex TripleTOF 5600^+^ quadrupole time-of-flight tandem mass spectrometer (Sciex, Redwood City, CA, USA). The TOF MS and collision-induced dissociation (CID) MS/MS data was acquired in positive electrospray ionization mode with dynamic background subtraction (DBS). Source parameters were optimized by infusing endogenous metabolites reference standard solution and defined as follows: temperature, 550 °C; ion spray voltage, 5500 V; nebulizer gas (Gas 1), 55 psi; heater gas (Gas 2), 55 psi; curtain gas, 35 psi; declustering potential, 100 V; and collision energy, 10 V. For IDA, any TOF MS survey scan peak exceeding 100 cps was selected for dependent scan, with isotopes within 4 Da being excluded. Mass tolerance was 50 mDa. 8 candidate ions were allowed per cycle. The collision energy was set to 35 V with a collision energy spread of 15 V. The TOF MS was acquired scanning a mass range from *m*/*z* 50–1000 followed by product ion scanning from *m*/*z* 50–1000 with 200 and 100 ms accumulation times, respectively. An automated calibration was run after every fifth injection. All operations and data acquisition were controlled by the Analyst TF 1.7 software (Sciex, Redwood City, CA, USA).

### 4.5. Data Processing and Multivariate Data Analysis

The LC-MS data files were imported into MasterView software (Version 1.1, Sciex, Redwood City, CA, USA). Chromatographic peak finding, peak alignment, peak filtering and data normalization were all achieved using MasterView software. Main filtering parameters were set as follows: minimum retention time, 0 min; minimum spectral peak width, 25 ppm; minimum RT peak width, 6 scans; noise threshold, 100; retention time tolerance, 0.5 min; mass tolerance, 5 ppm. The chromatographic peaks were normalized using total area sums. Isotope ions were excluded before statistical analysis, while Pareto scaling and log transformation was applied to the data processing before PCA-DA was performed. All the compounds with significance threshold which satisfying corrected *p*-value cut-off 0.05 in one-way ANOVA were considered as potential biomarkers. The software used for other statistical analysis was SPSS 19.0 unless specifically mentioned otherwise.

Multivariate analyses of log-transformed and Pareto-scaled brain homogenates and plasma metabolome data were performed using MetaboAnalyst 3.0 (Xia Lab at McGill University, Montreal, QC, Canada), a web-based metabolomics data analysis software, for further processing. PLS-DA was conducted to give the contribution of the metabolites by VIP score, while HCA to achieve cluster analysis, implemented in the MetaboAnalyst approach commonly used for unsupervised clustering, was constructed based on the potential candidates of importance.

### 4.6. Identification of Biomarkers and Metabolic Pathway

The identification of potential biomarkers was determined by LC-HRMS. Sciex PeakView 2.2 and MasterView 1.1 software (Sciex, Redwood City, CA, USA) was used to help confirm the accurate mass of both precursor and product ions after CID on the specific metabolites. Afterwards, compound identification continued by analyzing with the fragmentation patterns, chromatographic retention characteristics, as well as comparing the mass-spectra with the authentic standards. The accurate mass and fragmentation of the potential metabolites was matched with the metabolites from online databases, mzCloud (www.mzcloud.org), METLIN (www.metlin.scripps.edu/) and HMDB (www.hmdb.ca).

Pathway analysis using the KEGG pathway database was performed by MetaboAnalyst software for the identified potential metabolites with significant changes to identify the top headache-correlated metabolic pathways. Meanwhile, pathway topology analysis was used to generate the impact value of the relative metabolic pathways using a relative-betweenness centrality test. Cytoscape software was then used to construct a visualized metabolic network by connecting the potential metabolic pathways to facilitate the mechanism study of headache.

## 5. Conclusions

A comprehensive metabolomics profiling on the brain and plasma of NO-donor induced headache rats by tandem high resolution mass spectrometry along with multivariate data analysis was successfully established and integrally investigated. Both the brain and plasma metabolome were obviously altered after headache modelling and WZYD treatment, and the headache-relief effect by WZYD was then verified. After the global non-target metabolomics screening, a total of 17 metabolic biomarker candidates which may be related to headache attack were characterized and their correlative metabolomic pathways were revealed. In addition, it was speculated that the potential active targets of WZYD on headache may be more comprehensive than chemical treatments. The study demonstrated that the metabolomics strategy might serve as a powerful approach for investigating the mechanisms of headache and WZYD using the processing method, while providing solutions to explore the molecular basis of diseases and identify potential biomarkers.

## Figures and Tables

**Table 1:** Potential brain homogenates biomarkers detected by liquid chromatography-high resolution mass spectrometry (LC-HRMS) and their intensities in control, model, WZYD and flunarizine groups analyzed by one-way ANOVA.

RT	*m*/*z*	Compound	Peak Intensity (Mean ± SD)			
			Control	Model	WZYD	Flunarizine
0.81	258.1108	GPC	6.61 × 10^4^ ± 3.41 × 10^4^	1.13 × 10^5^ ± 5.40 × 10^4 ##^	2.17 × 10^4^ ± 4.03 × 10^3^ **	2.42 × 10^4^ ± 7.77 × 10^3^ **
0.82	175.1183	Arginine	1.68 × 10^5^ ± 4.88 × 10^4^	1.31 × 10^5^ ± 2.10 × 10^4 ##^	2.77 × 10^5^ ± 4.33 × 10^4^ **	1.91 × 10^5^ ± 2.29 × 10^4^ **
1.11	348.0707	AMP	3.14 × 10^5^ ± 2.05 × 10^5^	5.60 × 10^5^ ± 2.04 × 10^5 ##^	1.62 × 10^5^ ± 3.37 × 10^4^ **	1.82 × 10^5^ ± 6.37 × 10^4^ **
1.14	664.1144	NAD	5.89 × 10^4^ ± 2.29 × 10^4^	7.67 × 10^4^ ± 2.99 × 10^4 #^	2.29 × 10^4^ ± 4.99 × 10^3^ **	3.70 × 10^4^ ± 1.70 × 10^4^ **
1.18	308.0895	GSH	2.33 × 10^6^ ± 1.14 × 10^6^	3.06 × 10^6^ ± 1.09 × 10^6 #^	9.56 × 10^4^ ± 4.70 × 10^4^ **	6.50 × 10^4^ ± 2.42 × 10^4^ **
1.34	613.1597	GSSG	1.39 × 10^6^ ± 7.23 × 10^5^	1.72 × 10^6^ ± 4.48 × 10^5 #^	3.19 × 10^5^ ± 2.32 × 10^5^ **	2.32 × 10^5^ ± 1.81 × 10^5^ **
2.20	136.0616	Adenine	4.61 × 10^5^ ± 2.49 × 10^5^	6.69 × 10^5^ ± 1.75 × 10^5 ##^	2.74 × 10^5^ ± 4.40× 10^4^ **	2.08 × 10^5^ ± 2.54 × 10^4^ **
2.24	268.1025	Adenosine	2.70 × 10^6^ ± 2.37 × 10^6^	5.20 × 10^6^ ± 2.05 × 10^6 ##^	1.97 × 10^5^ ± 5.66 × 10^4^ **	2.70 × 10^5^ ± 1.41 × 10^5^ **
3.51	100.0764	Valerolactam	9.97 × 10^3^ ± 3.32 × 10^3^	2.19 × 10^4^ ± 2.18 × 10^4 ##^	1.02 × 10^4^ ± 3.93 × 10^3^ **	1.03 × 10^4^ ± 5.05 × 10^3^ **
6.56	232.1540	Butyrylcarnitine	5.87 × 10^4^ ± 3.35 × 10^4^	8.25 × 10^4^ ± 4.18 × 10^4 ##^	1.58 × 10^4^ ± 3.90 × 10^3^ **	2.58 × 10^4^ ± 8.02 × 10^3^ **
6.74	114.0914	Caprolactam	8.00 × 10^5^ ± 1.45 × 10^5^	8.98 × 10^5^ ± 1.31 × 10^5 ##^	2.17 × 10^5^ ± 1.26 × 10^4^ **	2.27 × 10^5^ ± 2.44 × 10^4^ **
20.38	385.3465	Cholecalciferol	1.82 × 10^5^ ± 8.48 × 10^4^	1.24 × 10^5^ ± 7.58 × 10^4 #^	1.75 × 10^5^ ± 5.27 × 10^4^ *	1.23 × 10^5^ ± 3.25 × 10^4^

^#^ *p* < 0.05 and ^##^ *p* < 0.01 compared with control group; * *p* < 0.05 and ** *p* < 0.01 compared with model group. GPC: glycerophosphocholine; AMP: adenosine monophosphate; NAD: nicotinamide adenine dinucleotide; GSH: glutathione; GSSG: oxidized glutathione.

**Table 2:** Potential plasma biomarkers detected by LC-HRMS and their intensities in control, model, WZYD and flunarizine groups analyzed by one-way ANOVA.

RT	*m*/*z*	Compound	Peak Intensity (Mean ± SD)			
			Control	Model	WZYD	Flunarizine
0.93	175.1188	Arginine	2.91 × 10^5^ ± 2.47 × 10^4^	2.28 × 10^5^ ± 4.79 × 10^4 ##^	2.60 × 10^5^ ± 3.45 × 10^4^ *	2.05 × 10^5^ ± 3.01 × 10^4^
0.94	162.1122	Carnitine	7.47 × 10^5^ ± 1.17 × 10^5^	5.87 × 10^5^ ± 8.45 × 10^4 ##^	6.87 × 10^5^ ± 1.65 × 10^5^ *	5.70 × 10^5^ ± 9.79 × 10^4^
3.12	192.0660	5-HIAA	4.63 × 10^4^ ± 1.07 × 10^4^	3.92 × 10^4^ ± 8.96 × 10^3 #^	4.24 × 10^4^ ± 9.62 × 10^3^	4.01 × 10^4^ ± 8.89 × 10^3^
6.64	132.0808	3-Methyl-indole	9.68 × 10^4^ ± 1.69 × 10^4^	8.37 × 10^4^ ± 1.04 × 10^4 #^	1.06 × 10^5^ ± 1.43 × 10^4^ **	8.39 × 10^4^ ± 1.22 × 10^4^
7.17	134.0592	Hydroxy-indole	7.31 × 10^4^ ± 3.61 × 10^4^	4.49 × 10^4^ ± 2.38 × 10^4 ##^	6.31 × 10^4^ ± 3.39 × 10^4^	3.65 × 10^4^ ± 1.43 × 10^4^
9.2	176.0700	IAA	2.03 × 10^4^ ± 1.06 × 10^4^	1.19 × 10^4^ ± 4.27 × 10^3 ##^	1.37 × 10^4^ ± 3.87 × 10^3^	8.95 × 10^3^ ± 2.36 × 10^3^

^#^ *p* < 0.05 and ^##^ *p* < 0.01 compared with control group; * *p* < 0.05 and ** *p* < 0.01 compared with model group. 5-HIAA: 5-hydroxyindoleacetic acid.

**Table 3:** Metabolic pathway analysis with MetaboAnalyst.

Pathway Name	Total	Expected	Hits	Raw *p*	Impact
Glutathione metabolism	26	0.25963	2	0.026253	0.39790
Purine metabolism	68	0.67903	3	0.027048	0.06056
Tryptophan metabolism	41	0.40942	2	0.060817	0.01473
Nicotinate and nicotinamide metabolism	13	0.12981	1	0.122810	0.20833
Lysine degradation	20	0.19971	1	0.182990	0.00000
Glycerophospholipid metabolism	30	0.29957	1	0.262330	0.02315
Arginine and proline metabolism	44	0.43937	1	0.361430	0.08228
Aminoacyl-tRNA biosynthesis	67	0.66904	1	0.497840	0.00000

## Supplementary Materials

The following are available online. Figure S1: TOF MS total ion chromatograms of representative brain homogenates samples from (A) control, (B) model, (C) WZDY and (D) flunarizine group; and plasma samples from (E) control, (F) model, (G) WZDY and (H) flunarizine group in positive ion mode; Figure S2: Extracted ion chromatograms of the potential metabolite biomarkers found in both brain and plasma metabolomics, (A) GPC, (B) Arginine, (C) AMP, (D) NAD, (E) GSH, (F) GSSG, (G) Adenine, (H) Adenosine, (I) Valerolactam, (J) Butyrylcarnitine, (K) Caprolactam and (L) Cholecalciferol in brain; (M) Arginine, (N) Carnitine, (O) 5-HIAA, (P) 3-Methylindole, (Q) Hydroxyindole and (R) IAA in plasma; Figure S3: VIP scores analysis based on weighted coefficients of the PLS-DA model used to rank the contribution of the metabolites to the discrimination between the four groups; Figure S4: Integrated metabolic network in headache subjects. Altered metabolites were mapped to KEGG and SMPD reference pathways and interaction networks were generated in Cytoscape. The network nodes (blue and pink) represent pathways and related metabolites detected, respectively; Table S1: Comparison of the times of head-scratching of the rats (*n* = 15) in 2 h; Table S2: The database information of the potential biomarkers and their trends in headache rat brain tissue; Table S3: The database information of the potential biomarkers and their trends in headache rat plasma; Table S4: Principle metabolic pathways of the metabolites in KEGG database.

## Abbreviations

5-HIAA	5-hydroxyindoleacetic acid
5-HT	5-hydroxytryptamine
AMP	adenosine monophosphate
CID	collision induced dissociation
CNS	central nervous system
DBS	dynamic background subtraction
GPC	glycerophosphocholine
GSH	glutathione
GSSG	oxidized glutathione
HCA	hierarchical cluster analysis
HDAC	histone deacetylases
IAA	indoleacetic acid
IDA	information dependent acquisition
LC-HRMS	liquid chromatography-high resolution mass spectrometry
NAD	nicotinamide adenine dinucleotide
NO	nitric oxide
NTG	nitroglycerin
PCA-DA	principal component analysis–discriminant analysis
PLS-DA	partial least squares-discriminant analysis
TCM	Traditional Chinese medicine
QTOF	quadrupole time-of-flight
WZYD	Wu-Zhu-Yu decoction

## References

[1] Odaguchi H., Wakasugi A., Ito H., Shoda H., Gono Y., Sakai F., Hanawa T. (2006). The efficacy of goshuyuto, a typical Kampo (Japanese herbal medicine) formula, in preventing episodes of headache. Curr. Med. Res. Opin..

[2] Schwedt T.J. (2014). Chronic migraine. BMJ.

[3] Moskowitz M.A., Buzzi M.G. (2010). Migraine general aspects. Handb. Clin. Neurol..

[4] World Health Organization (WHO) (2016). Headache disorders. Fact Sheet No. 277.

[5] Goadsby P.J. (2009). The vascular theory of migraine—A great story wrecked by the facts. Brain J. Neurol..

[6] Brennan K.C., Charles A. (2010). An update on the blood vessel in migraine. Curr. Opin. Neurol..

[7] Goadsby P.J., Lipton R.B., Ferrari M.D. (2002). Migraine—Current understanding and treatment. N. Engl. J. Med..

[8] Olesen J. (2006). The Headaches.

[9] Gasparini C.F., Smith R.A., Griffiths L.R. (2017). Genetic and biochemical changes of the serotonergic system in migraine pathobiology. J. Headache Pain.

[10] Zhang A., Liu Q., Zhao H., Zhou X., Sun H., Nan Y., Zou S., Ma C.W., Wang X. (2016). Phenotypic characterization of nanshi oral liquid alters metabolic signatures during disease prevention. Sci. Rep..

[11] Chu H., Zhang A., Han Y., Lu S., Kong L., Han J., Liu Z., Sun H., Wang X. (2016). Metabolomics approach to explore the effects of Kai-Xin-San on Alzheimer’s disease using UPLC/ESI-Q-TOF mass spectrometry. J. Chromatogr. B.

[12] Lionetto L., Gentile G., Bellei E., Capi M., Sabato D., Marsibilio F., Simmaco M., Pini L.A., Martelletti P. (2013). The omics in migraine. J. Headache Pain.

[13] Cao H., Zhang A., Sun H., Zhou X., Guan Y., Liu Q., Kong L., Wang X. (2015). Metabolomics-proteomics profiles delineate metabolic changes in kidney fibrosis disease. Proteomics.

[14] Nan Y., Zhou X., Liu Q., Zhang A., Guan Y., Lin S., Kong L., Han Y., Wang X. (2016). Serum metabolomics strategy for understanding pharmacological effects of ShenQi pill acting on kidney yang deficiency syndrome. J. Chromatogr. B.

[15] Xia J., Wishart D.S. (2016). Using MetaboAnalyst 3.0 for Comprehensive Metabolomics Data Analysis. Curr. Protoc. Bioinform..

[16] MetaboAnalyst—A Comprehensive Tool for Metabolomics Analysis and Interpretation. http://www.metaboanalyst.ca.

[17] Gokce Cokal B., Aytac B., Durak Z.E., Gunes H.N., Ozturk B., Keskin Guler S., Durak I., Yoldas T.K. (2015). Serum oxidant and antioxidant status of patients with chronic tension-type headache: Possible effects of medical treatment. Neurol. Sci. Off. J. Ital. Neurol. Soc. Ital. Soc. Clin. Neurophysiol..

[18] Srikiatkhachorn A. (2001). Pathophysiology of chronic daily headache. Curr. Pain Headache Rep..

[19] Rogers K.L., Grice I.D., Griffiths L.R. (2000). Inhibition of platelet aggregation and 5-HT release by extracts of Australian plants used traditionally as headache treatments. Eur. J. Pharm. Sci. Off. J. Eur. Fed. Pharm. Sci..

[20] Zwilling D., Huang S.Y., Sathyasaikumar K.V., Notarangelo F.M., Guidetti P., Wu H.Q., Lee J., Truong J., Andrews-Zwilling Y., Hsieh E.W. (2011). Kynurenine 3-monooxygenase inhibition in blood ameliorates neurodegeneration. Cell.

[21] Zadori D., Klivenyi P., Vamos E., Fulop F., Toldi J., Vecsei L. (2009). Kynurenines in chronic neurodegenerative disorders: Future therapeutic strategies. J. Neural Transm..

[22] Bynoe M.S., Viret C., Yan A., Kim D.G. (2015). Adenosine receptor signaling: A key to opening the blood-brain door. Fluids Barriers CNS.

[23] Berger F., Ramirez-Hernandez M.H., Ziegler M. (2004). The new life of a centenarian: Signalling functions of NAD(P). Trends Biochem. Sci..

[24] Corda D., Di Girolamo M. (2003). Functional aspects of protein mono-ADP-ribosylation. EMBO J..

[25] Tarighat Esfanjani A., Mahdavi R., Ebrahimi Mameghani M., Talebi M., Nikniaz Z., Safaiyan A. (2012). The effects of magnesium, L-carnitine, and concurrent magnesium-L-carnitine supplementation in migraine prophylaxis. Biol. Trace Elem. Res..

[26] Prakash S., Mehta N.C., Dabhi A.S., Lakhani O., Khilari M., Shah N.D. (2010). The prevalence of headache may be related with the latitude: A possible role of Vitamin D insufficiency?. J. Headache Pain.

[27] Kjaergaard M., Eggen A.E., Mathiesen E.B., Jorde R. (2012). Association between headache and serum 25-hydroxyvitamin D: The Tromso Study: Tromso 6. Headache.

[28] Turner M.K., Hooten W.M., Schmidt J.E., Kerkvliet J.L., Townsend C.O., Bruce B.K. (2008). Prevalence and clinical correlates of vitamin D inadequacy among patients with chronic pain. Pain Med..

[29] Xu H., Li Q., Yin Y., Lv C., Sun W., He B., Liu R., Chen X., Bi K. (2013). Simultaneous determination of three alkaloids, four ginsenosides and limonin in the plasma of normal and headache rats after oral administration of Wu-Zhu-Yu decoction by a novel ultra fast liquid chromatography-tandem mass spectrometry method: Application to a comparative pharmacokinetics and ethological study. J. Mass Spectrom..

