# Supplementary material for: Metabolomics Strategy Using High Resolution Mass Spectrometry Reveals Novel Biomarkers and Pain-Relief Effect of Traditional Chinese Medicine Prescription Wu-Zhu-Yu Decoction Acting on Headache Modelling Rats
Source: Molecules. 2017 Dec 18;22(12):2110. doi: 10.3390/molecules22122110 (PMC6149820; doi:10.3390/molecules22122110)
Supplement: Supplementary file 1 [file molecules-22-02110-s001.zip › Supplementary Materials - Method Validation.pdf]

Analytical method validation

The instrumental precision, sample repeatability and within-day stability were determined prior to the analysis of experimental samples. Quality control (QC) samples were obtained by mixing an equal amount (100 μl of brain homogenates or 10 μl of plasma) from each sample. The extracted ion chromatographic peaks of six ions (*m/z* 132.0762_0.94 min, *m/z* 137.0447_1.28 min, *m/z* 205.0979_6.70 min, *m/z* 161.0946_9.30 min, *m/z* 327.0093_12.53 min, *m/z* 526.2928_13.67 min) from brain samples and six ions (*m/z* 112.0498_ 1.13 min, *m/z* 205.0961_ 6.65 min, *m/z* 531.7684_ 7.73 min, *m/z* 318.2992_ 11.41 min, *m/z* 496.3388_ 14.52 min, *m/z* 524.3700_ 16.77 min) from plasma samples were selected for method validation. The selected ions were distributed over the analysis *m/z* and time range. The instrumental precision was validated by analyzing five injections continuously of the same QC sample. Then five aliquots of a random sample were used to investigate the sample repeatability. The within-day stability was evaluated using five injections of the same QC sample at 0, 4, 8, 12 and 24 h. The results (RSD%) of the retention times were all not more that 5% and the intensities were not more than 10%. The method validation results confirmed the reliability of the method.
