# Supplementary material for: Metabolomics Strategy Using High Resolution Mass Spectrometry Reveals Novel Biomarkers and Pain-Relief Effect of Traditional Chinese Medicine Prescription Wu-Zhu-Yu Decoction Acting on Headache Modelling Rats
Source: Molecules. 2017 Dec 18;22(12):2110. doi: 10.3390/molecules22122110 (PMC6149820; doi:10.3390/molecules22122110)
Supplement: Supplementary file 1 [file molecules-22-02110-s001.zip › Tables S1-4.pdf]

molecules

Table S1 Comparison of the times of head-scratching of the rats (n=15) in 2 h.

Groups	Times (mean ± SD)	*p*
A Control	3.8 ± 5.5	---
B Model	465.5 ± 400.1 **	0.000 [a]
C WZY Decoction	216.7 ± 119.7 **	0.000 [b]
D Flunarizine	220.3 ± 183.8 **	0.000 [b]

a: compared to group A; b: compared to group B; **p<0.01.

Table S2 The database information of the potential biomarkers and their trends in headache rat brain tissue.

NO	Metabolite	Formula	HMDB	METLIN	KEGG	Trend in Model
1	Glycerophosphocholine (GPC)	$C_8H_{20}NO_6P$	HMDB00086	370	C00670	↑
2	Arginine	$C_6H_{14}N_4O_2$	HMDB00517	13	C00062	↓
3	Adenosine monophosphate (AMP)	$C_{10}H_{14}N_5O_7P$	HMDB03540	63401	C01367	↑
4	Nicotinamide adenine dinucleotide (NAD)	$C_{21}H_{27}N_7O_{14}P_2$	HMDB00902	101	C00003	↑
5	Glutathione (GSH)	$C_{10}H_{17}N_3O_6S$	HMDB00125	44	C00051	↑
6	Oxidized glutathione (GSSG)	$C_{20}H_{32}N_6O_{12}S_2$	HMDB03337	45	C00127	↑
7	Adenine	$C_5H_5N_5$	HMDB00034	85	C00147	↑
8	Adenosine	$C_{10}H_{13}N_5O_4$	HMDB00050	86	C00212	↑
9	Valerolactam	C_5H_9NO	HMDB11749	62467	-	↑
10	Butyrylcarnitine	$C_{11}H_{21}NO_4$	HMDB02013	964	C02862	↑
11	Caprolactam	$C_6H_{11}NO$	-	44753	C06593	↑
12	Cholecalciferol (Vitamin D3)	$C_{27}H_{44}O$	HMDB14315	165	C05443	↓

Table S3 The database information of the potential biomarkers and their trends in headache rat plasma.

NO	Metabolite	Formula	HMDB	METLIN	KEGG	Trend in Model
1	Arginine	$C_6H_{14}N_4O_2$	HMDB00517	13	C00062	↓
2	Carnitine	$C_7H_{15}NO_3$	HMDB00062	52	C00318	↓
3	5-Hydroxyindoleacetic acid (5-HIAA)	$C_{10}H_9NO_3$	HMDB00763	2975	C05635	↓
4	3-Methylindole	C_9H_9N	HMDB00466	5453	C08313	↓
5	Hydroxyindole	C_8H_7NO	HMDB59805	34514	C02040	↓
6	Indoleacetic acid (IAA)	$C_{10}H_9NO_2$	HMDB00197	70	C00954	↓

Table S4 Principle metabolic pathways of the metabolites in KEGG database.

NO	Metabolite	Pathways
1	Arginine	Arginine and proline metabolism; Arginine biosynthesis; Aminoacyl-tRNA biosynthesis
2	Glutathione	Glutathione metabolism; Cysteine and methionine metabolism
3	Oxidized glutathione	Glutathione metabolism
4	Indoleacetic acid	Tryptophan metabolism
5	5-Hydroxyindoleacetic acid	Tryptophan metabolism
6	3-Methylindole	-
7	Hydroxyindole	-
8	Adenine	Purine metabolism
9	Adenosine	Purine metabolism; cAMP signaling pathway; Sphingolipid signaling pathway
10	Adenosine monophosphate	Purine metabolism
11	Nicotinamide adenine dinucleotide	Nicotinate and nicotinamide metabolism; Oxidative phosphorylation; Vitamin digestion and absorption
12	Carnitine	Lysine degradation; Bile secretion
13	Butyrylcarnitine	-
14	Glycerophosphocholine	Glycerophospholipid metabolism; Ether lipid metabolism
15	Caprolactam	Caprolactam degradation
16	Valerolactam	Valerolactam degradation
17	Cholecalciferol	Vitamin digestion and absorption
