# Supplementary figures and images for: Metabolomics Strategy Using High Resolution Mass Spectrometry Reveals Novel Biomarkers and Pain-Relief Effect of Traditional Chinese Medicine Prescription Wu-Zhu-Yu Decoction Acting on Headache Modelling Rats
Source: Molecules. 2017 Dec 18;22(12):2110. doi: 10.3390/molecules22122110 (PMC6149820; doi:10.3390/molecules22122110)

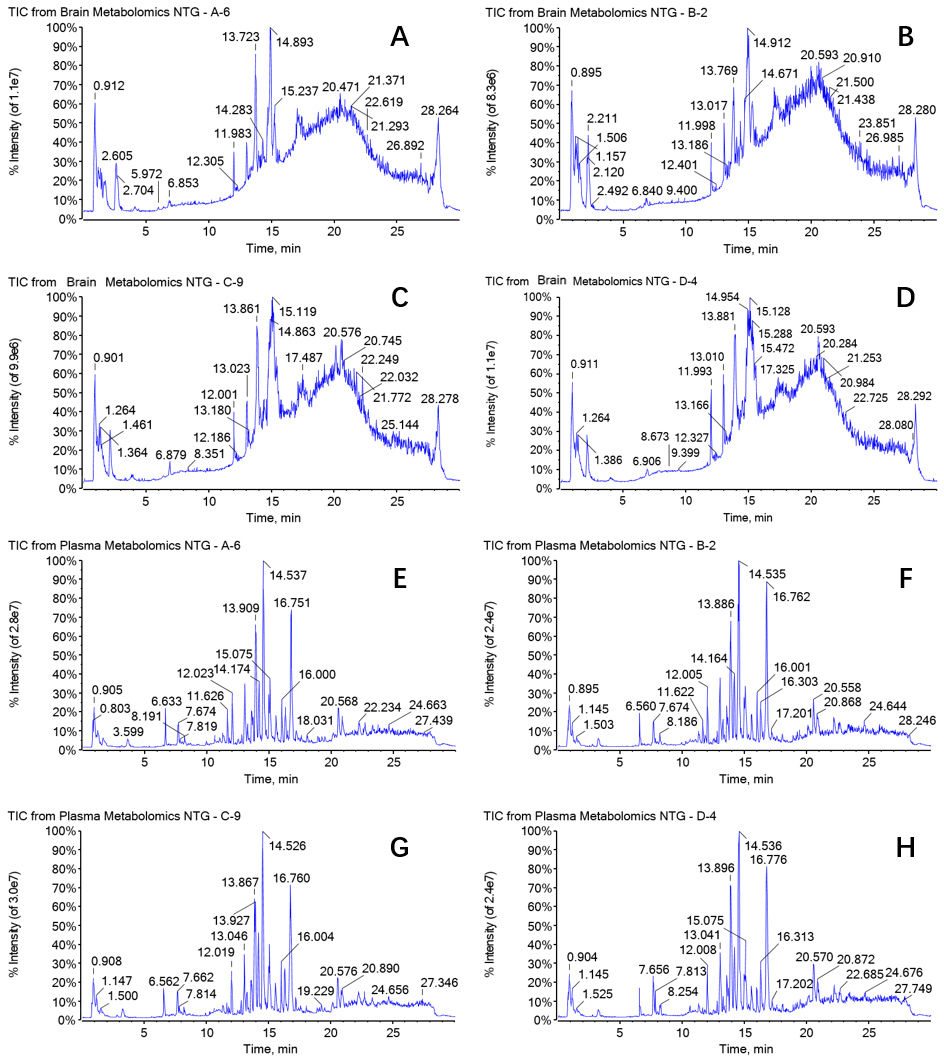

Supplement: Supplementary file 1 [file molecules-22-02110-s001.zip › Figure S1.tif]

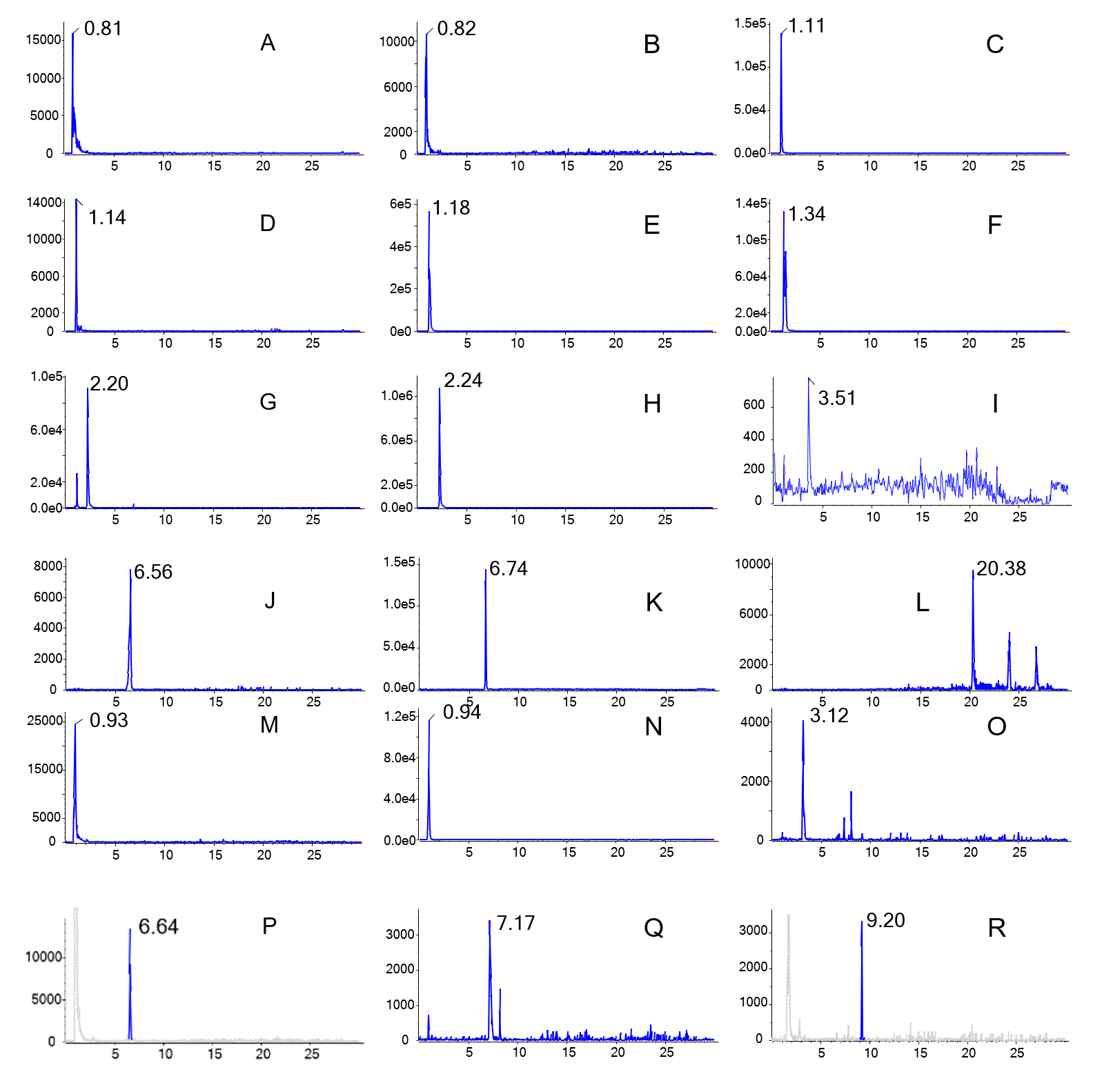

Supplement: Supplementary file 1 [file molecules-22-02110-s001.zip › Figure S2.tif]

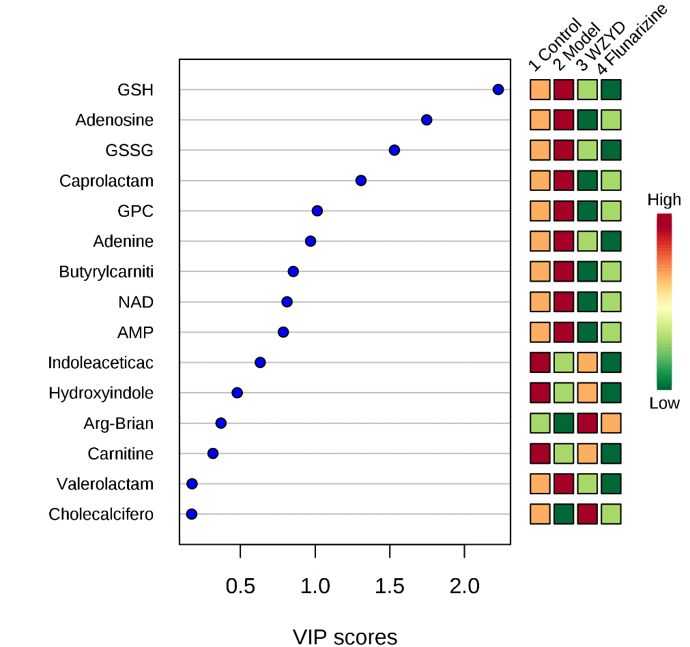

Supplement: Supplementary file 1 [file molecules-22-02110-s001.zip › Figure S3.tif]

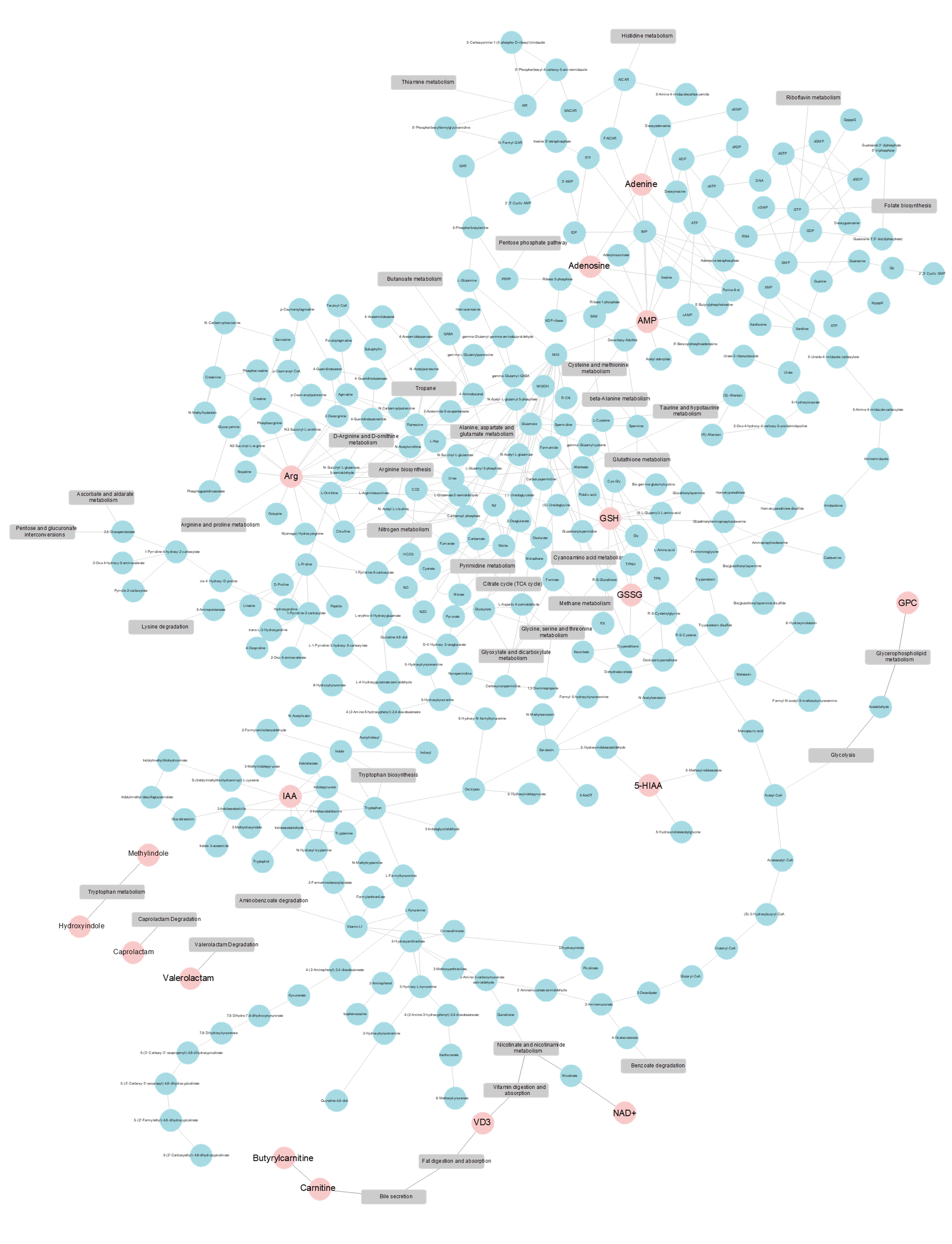

Supplement: Supplementary file 1 [file molecules-22-02110-s001.zip › Figure S4.tif]
